# A cortex-specific penicillin-binding protein contributes to heat resistance in *Clostridioides difficile* spores

**DOI:** 10.1016/j.anaerobe.2021.102379

**Published:** 2021-08

**Authors:** Yasir Adil Jabbar Alabdali, Peter Oatley, Joseph A. Kirk, Robert P. Fagan

**Affiliations:** Florey Institute, Department of Molecular Biology and Biotechnology, University of Sheffield, UK

**Keywords:** *Clostridioides difficile*, Sporulation, SpoVD, Peptidoglycan, Penicillin-binding protein

## Abstract

**Background:**

Sporulation is a complex cell differentiation programme shared by many members of the Firmicutes, the end result of which is a highly resistant, metabolically inert spore that can survive harsh environmental insults. *Clostridioides difficile* spores are essential for transmission of disease and are also required for recurrent infection. However, the molecular basis of sporulation is poorly understood, despite parallels with the well-studied *Bacillus subtilis* system. The spore envelope consists of multiple protective layers, one of which is a specialised layer of peptidoglycan, called the cortex, that is essential for the resistant properties of the spore. We set out to identify the enzymes required for synthesis of cortex peptidoglycan in *C. difficile*.

**Methods:**

Bioinformatic analysis of the *C. difficile* genome to identify putative homologues of *Bacillus subtilis spoVD* was combined with directed mutagenesis and microscopy to identify and characterise cortex-specific PBP activity.

**Results:**

Deletion of CDR20291_2544 (SpoVD_Cd_) abrogated spore formation and this phenotype was completely restored by complementation *in cis*. Analysis of SpoVD_Cd_ revealed a three domain structure, consisting of dimerization, transpeptidase and PASTA domains, very similar to *B. subtilis* SpoVD. Complementation with SpoVD_Cd_ domain mutants demonstrated that the PASTA domain was dispensable for formation of morphologically normal spores. SpoVD_Cd_ was also seen to localise to the developing spore by super-resolution confocal microscopy.

**Conclusions:**

We have identified and characterised a cortex specific PBP in *C. difficile*. This is the first characterisation of a cortex-specific PBP in *C. difficile* and begins the process of unravelling cortex biogenesis in this important pathogen.

## Highlights

• CDR20291_2544 encodes a *C. difficile* homologue of the *B subtilis* SpoVD.
• Mutation of *spoVD*_*Cd*_ completely prevents the formation of heat-resistant spores.
• The SpoVD_Cd_ PASTA domain was dispensable for its function.
• SpoVD_Cd_ localises to the developing spore.

## 1 Introduction

*C. difficile* is the most common cause of nosocomial antibiotic-associated diarrhoea, with an estimated 453,000 infections and 29,300 deaths per year in the USA alone [1]. *C. difficile* infection (CDI) requires prior disruption to the gut microbiota, most commonly due to an administered antibiotic [2]. As current treatments largely rely on antibiotic therapy, with further consequent damage to the microbiota, recurrent disease is common and is associated with worse patient prognosis [3]. In recent years there have been dramatic changes in *C. difficile* epidemiology, in particular due to the emergence of the epidemic ribotype 027 lineage, a previously rare ribotype that was responsible for a series of large hospital outbreaks in North America in the early years of this century before spreading worldwide [4].

The spore is an absolute requirement for transmission of disease [5], since it allows the organism to transit the lethal aerobic environment while also providing significant resistance to desiccation, heat and common disinfectants [6]. As a result, the spores shed by an infected individual can survive in the environment for an extended period of time. This environmental contamination is a particular problem in hospital environments where large numbers of susceptible individuals are housed in close proximity. The process of sporulation is still relatively poorly understood, despite significant advances in recent years [7]. We have previously used high-density transposon mutagenesis and TraDIS to identify a subset of *C. difficile* genes required for formation of mature heat-resistance spores [8]. In total, transposon insertions in 798 genes were found to significantly impact sporulation, many with no clear homology to previously characterised proteins. Very few of these 798 genes have been studied in *C. difficile* but many have homologues in the well-studied *Bacillus subtilis* sporulation pathway. However, despite the clear parallels between sporulation in *B. subtilis* and *C. difficile*, there are enough critical differences to greatly reduce the value of assumptions based on homology [[9], [10], [11]]. The response regulator Spo0A is the master regulator of sporulation, phosphorylation of which sets in motion a complex asymmetric cell differentiation programme involving the sequential activation of a series of dedicated sigma factors that are in turn responsible for the expression of the individual regulons required for correct spore morphogenesis [12]. The result is the complex multi-layered spore structure that lends robustness to environmental insult. The spore consists of a dehydrated core surrounded by a membrane and peptidoglycan cell wall (primordial wall) derived from the mother cell envelope. Around this is a thick peptidoglycan cortex, synthesised during spore maturation, and a second membrane, formed as a result of engulfment of the prespore by the mother cell. The outer surface consists of multiple layers of highly crosslinked proteins. The order and timing of synthesis of each of these layers is critical and disruption to any of the steps typically results in the formation of defective spores that lack full resistance properties [9].

In *B. subtilis* the peptidoglycan of the primordial wall and cortex differ in structure allowing differentiation by the cortex lytic hydrolases during germination [13]. The primordial cell wall consists of typical alternating β-1→4-linked N-acetyl glucosamine and N-acetyl muramic acid residues, crosslinked by 4–3 linked peptide stems attached to the muramic acid moieties. In cortex peptidoglycan, every second N-acetyl muramic acid is modified to muramic-δ-lactam, resulting in fewer stem peptides, fewer crosslinks and a more flexible overall structure [14]. The class B penicillin-binding protein (PBP) SpoVD is critical for synthesis of *B. subtilis* cortex [15]. During sporulation SpoVD is expressed in the mother cell where it interacts with the SEDS protein SpoVE to enable localisation to the asymmetric division septum [16]. An N-terminal transmembrane alpha helix anchors the protein in the membrane, placing the majority of the protein in the inter-membrane space where the cortex is ultimately synthesised [17]. SpoVD consists of a PBP dimerization domain, followed by a transpeptidase domain and a penicillin-binding protein and serine/threonine kinase associated (PASTA) domain, the last of which is dispensable for cortex formation [18].

*C. difficile* vegetative cell peptidoglycan is superficially similar to that of *B. subtilis*, albeit with a preponderance of 3-3 cross-linking as a result of l,d-transpeptidase activity [19]. The structure of the *C. difficile* cortex peptidoglycan also differs, with only approximately half the abundance of muramic-δ-lactam modifications and significant GlcNAc *N*-deacetylation that is not seen at all in *B. subtilis* [20,21]. Although the enzymes required for cortex synthesis have yet to be characterised in detail, we have previously shown that CDR20291_2544 is required for sporulation [8] and it has been recently been confirmed that this enzyme is required for sporulation and is the target of cephamycin antibiotics that inhibit spore formation [22]. Here we set out to identify and characterise the major *C. difficile* cortex PBP.

## 2 Methods

### 2.1 Bacterial strains and growth conditions

All bacterial strains, plasmids and oligonucleotides used in this study are described in Table 1. *E. coli* strains were routinely grown in LB broth and on LB agar, while *C. difficile* strains were grown in TY broth [23] and on brain heart infusion agar. Cultures were supplemented with chloramphenicol (15 μg/ml), thiamphenicol (15 μg/ml) or cycloserine (250 μg/ml) as appropriate.

**Table 1:** Strains, plasmids and oligonucleotides used in this study.

Strain	Characteristics	Source
R20291	*C. difficile* ribotype 027 strain isolated during an outbreak at Stoke Mandeville hospital, UK in 2006.	[34]
R20291Δ*pyrE*	An R20291 *pyrE* deletion mutant.	[26]
R20291Δ*spoVD*	R20291 with the entire *spoVD* gene, except the first and last three codons, deleted.	This study
R20291Δ*spoVD pyrE::spoVD*	R20291Δ*spoVD* complemented by simultaneous restoration of the wild type *pyrE* gene and insertion of *spoVD* under the native promoter between *pyrE* and the downstream R20291_0189.	This study
R20291 *SNAP*-*spoVD*	R20291 with the native *spoVD* locus modified by homologous recombination to add the coding sequence of SNAP to the 5′ end of *spoVD*.	This study
CA434	*E. coli* conjugative donor. HB101 carrying R702.	[35]
NEB5α	*fhuA2 Δ(argF-lacZ)U169 phoA glnV44 Φ80Δ (lacZ)M15 gyrA96 recA1 relA1 endA1 thi-1 hsdR17.*	New England Biolabs
**Plasmid**	**Characteristics**	**Source**
pMTL960	*E. coli*-*C. difficile* shuttle vector.	Nigel Minton
pRPF150	P_*cwp2*_-*Strep*-tag II-*secA2* cassette cloned between KpnI and BamHI sites in pMTL960.	[36]
pJAK012	pRPF150 modified to introduce an XhoI site between *Strep*-tag II encoding sequence and the *secA2* gene.	This study
pJAK032	Strep Tag II coding sequence in pJAK012 replaced with a codon-optimized *CLIP* gene.	This study
pFT46	Plasmid containing a *C. difficile* codon-optimized copy of the *SNAP* gene under the control of a tetracycline inducible promoter.	[37]
pMTL-YN4	Allele exchange vector for *pyrE*-based selection.	[26]
pMTL-YN2C	*pyrE* restoration vector allowing simultaneous insertion of cargo DNA between *pyrE* and R20291_0189.	[26]
pMTL-SC7215	Allele exchange vector for *codA*-based selection.	[25]
pYAA024	*spoVD* deletion: 1200 bp homology arms representing the sequence upstream and downstream of R20291_2544 (*spoVD*) cloned into pMTL-YN4. Designed to delete all but the first and last 9 bp of *spoVD*.	This study
pYAA027	SpoVD complementation: *spoVD* and its native promoter cloned into pMTLYN2C.	This study
pYAA031	Constitutive CLIP-SpoVD: *spoVD* cloned between XhoI and BamHI sites in pJAK032.	This study
pYAA047	SNAP-SpoVD: 1200 bp upstream of *spoVD* was fused to the coding sequence of SNAP and the first 1200 bp of *spoVD* and the subsequent recombination cassette cloned into pMTL-SC7215.	This study
pYAA048	SpoVD(ΔDimerization): pYAA031 modified by deletion of the sequence encoding the SpoVD PBP dimerization domain.	This study
pYAA049	SpoVD(ΔPASTA): pYAA031 modified by deletion of the sequence encoding the SpoVD PASTA domain.	This study
pYAA050	SpoVD(ΔTranspeptidase): pYAA031 modified by deletion of the sequence encoding the SpoVD transpeptidase domain.	This study
pYAA051	SpoVD(ΔDimer & PASTA): pYAA031 modified by deletion of the sequence encoding the SpoVD PBP dimerization and PASTA domains.	This study
pYAA052	His-SpoVD: *spoVD* cloned into pET-28a between NcoI and XhoI sites.	This study
**Oligonucleotide**	**Sequence**	**Use**
NF1957	GAGTCAGTTATAGATTCGATACTTGAC	To introduce an XhoI site into pRPF150 by inverse PCR with NF1958
NF1958	GAGTTTTTCAAATTGTGGATGACTCCAC	To introduce an XhoI site into pRPF150 by inverse PCR with NF1957
RF20	AAACTCCTTTTTGATAATCTCATGACC	To linearize pMTL-SC7215 with RF311
RF139^a^	GTCAGAGCTCGTTCTTTATTTAGATTAAATAAAGTCAATG	To clone *spoVD* into pMTL-YN4 with RF187
RF187^a^	GTCAGGATCCCTTAGGAATCAGAGAGTAGATAG	To clone *spoVD* into pMTL-YN4 with RF139
RF226	GATCGAGCTCGGAGGAACTACTATGGATAAAGATTGTGAAATGAAAAG	To add a 5′ SacI site onto a codon optimized *clip* gene fragment
RF227	GATCCTCGAGAGCAGCTGCTCCTAATCCTGGTTTTCCTAATC	To add 3xAla codons and a 3′ XhoI site onto a codon optimized *clip* gene fragment
RF311	TAGGGTAACAAAAAACACCG	To linearize pMTL-SC7215 with RF20
RF323^a^	GTCAGGATCCGTTTATGGGTATATGTTAATTATCTGTTAC	To clone R20291_2545 and *spoVD* into pMTL-YN2C with RF324
RF324^a^	GTCAGAGCTCCTTAGGAATCAGAGAGTAGATAG	To clone R20291_2545 and *spoVD* into pMTL-YN2C with RF323
RF374^a^	GATCCTCGAGAGAAAAGTAAAGAGGATAAGTAAGAAAAGG	To clone *spoVD* into pJAK032 with RF375
RF375^a^	GTCAGGATCCTTAGTTTTCAAAATATAGGGTTATACTTGAG	To clone *spoVD* into pJAK032 with RF374
RF461	CTCAAATCTATTCCCCCTAGTTATCC	To amplify *spoVD* promoter probe with RF462 for Southern blotting
RF462	GAATCTATGTGGTTATTCAAAAATCTCG	To amplify *spoVD* promoter probe with RF462 for Southern blotting
RF528	aaatacggtgttttttgttaccctaagtttAAGCTAGAATAGATGGACC	To amplify 1200 bp homology arm upstream of *spoVD*
RF529	acaatctttatccatATCTATTCCCCCTAGTTATCC	To amplify 1200 bp homology arm upstream of *spoVD*
RF530	ctagggggaatagatATGGATAAAGATTGTGAAATGAAGAGAACCAC	To amplify *SNAP*
RF531	cctctttacttttctAGCAGCTGCCCCAAGTCC	To amplify *SNAP*
RF532	cttggggcagctgctAGAAAAGTAAAGAGGATAAGTAAGAAAAG	To amplify first 1200 bp of *spoVD*
RF533	tttggtcatgagattatcaaaaaggagtttTAAATCTATACCTGTCTTATCCATAAG	To amplify first 1200 bp of *spoVD*
RF582	TATATCTCTTGTTTGTTGTTCTAGTGCTTTTG	To delete the coding sequence of the SpoVD PBP Dimerization domain with RF583
RF583	GCAAAAAAGGTTACTGCAATAGCTATG	To delete the coding sequence of the SpoVD PBP Dimerization domain with RF582
RF584	GGTTTAACTCCCAAATATTTTAAAGAGTCATTC	To delete the coding sequence of the SpoVD PASTA domain with RF585
RF585	TAAGGATCCACTAGTAACGGCC	To delete the coding sequence of the SpoVD PASTA domain with RF584
RF586	AGTATATAAAGAAGAAGAAAAAGCTGAGTATG	To delete the coding sequence of the SpoVD Transpeptidase domain with RF587
RF587	ATTATTTAACTCATAAGCTTTCTGTACTGC	To delete the coding sequence of the SpoVD Transpeptidase domain with RF586

a Restriction endonuclease sites are underlined.

### 2.2 Molecular biology methods

Routine molecular biology techniques were performed according to the manufacturers protocols except where otherwise stated. PCR using Phusion High-Fidelity DNA Polymerase, plasmid isolation and purification of DNA fragments were performed using kits and reagents supplied by Thermo Fisher Scientific according to the manufacturer's instructions. Restriction digestion, ligation and Gibson assembly were performed with enzymes supplied by New England Biolabs. Competent *E. coli* were transformed using standard methods and plasmid DNA was transferred to *C. difficile* as described previously [24]. *C. difficile* mutants were constructed by homologous recombination as described previously [25,26]. Mutants were confirmed by PCR and Southern blotting using the Amersham ECL Direct Labelling and Detection System kit (GE) according to the manufacturer's instructions. A 230 bp probe to the region immediately upstream of *spoVD*_*Cd*_ was generated by PCD using primer pair RF461/RF462.

### 2.3 Plasmid construction

pJAK032: pRPF150 was modified by inverse PCR using primer pair NF1957/NF1958 to introduce an XhoI site between the Strep Tag II and SecA2 coding sequences, yielding pJAK012. The Strep Tag II coding sequence was then excised using SacI and XhoI and replaced with a synthetic DNA fragment (IDT gBlock) consisting of a codon-optimized *CLIP* gene, modified by PCR with primer pair RF226/RF227 to add appropriate SacI and XhoI sites.

pYAA024: Homology arms upstream and downstream of *spoVD*_*Cd*_ were amplified by PCR using oligonucleotide pairs RF68/RF139 and RF69/RF187. The resulting PCR products were joined together in a SOEing PCR reaction and cloned between the BamHI and SacI sites in pMTL-YN4.

pYAA027: *spoVD*_*Cd*_ expression appears to be driven from a promoter upstream of CDR20291_2545. In order to ensure complementation at wild type levels a fragment comprising 282 bp upstream of CDR20291_2545, CDR20291_2545 itself and *spoVD*_*Cd*_ was amplified by PCR using primer pair RF324/RF325 and cloned between BamHI and SacI sites in pMTL-YN2C.

pYAA031: *secA2* in pJAK032 was replaced by *spoVD*_*Cd*_. *spoVD*_*Cd*_ was amplified by PCR using primer pair RF374/RF375, digested with BamHI and XhoI and ligated to pJAK032 backbone cut with the same enzymes.

pYAA047: 1200 bp upstream of *spoVD*_*Cd*_*,* the *SNAP* tag gene from pFT46 and the first 1200 bp of *spoVD*_*Cd*_ were amplified by PCR using primer pairs, RF528/RF529, RF530/RF531 and RF532/RF533 respectively. pMTL-SC7215 was linearized by PCR using primer pair RF20/RF311. The four DNA fragments were then joined in a Gibson assembly reaction.

pYAA048-050: The coding sequence of the SpoVD_Cd_ PBP dimerization domain (pYAA048; primers RF582/RF583), PASTA domain (pYAA049; primers RF584/RF585), or transpeptidase domain (pYAA050; primers RF586/RF587) were deleted by modification of pYAA031 by inverse PCR and subsequent recircularization by ligation.

pYAA051: pYAA048 was further modified to delete the coding sequence of the PASTA domain by inverse PCR with primers RF584/RF585.

### 2.4 Sporulation efficiency analysis

Overnight cultures of *C. difficile* R20291 were diluted in BHI broth to an OD_600nm_ of 0.01, incubated for 8 h at 37 °C, diluted to an OD_600nm_ of 0.0001 and finally incubated overnight. This allowed us to obtain cultures in stationary phase with no detectable spores (T = 0). This culture was then incubated for 5 days with vegetative cells and spores enumerated daily. For total viable counts, 10-fold serial dilutions were spotted onto BHIS agar supplemented with 0.1% sodium taurocholate. For total spore counts, the same process was carried out following a 30 min incubation at 65 °C. Colonies were counted after 24 h incubation at 37 °C and the assay was completed in biological triplicates. Formation of phase bright spores was also followed by phase-contrast microscopy at each time point. Samples fixed in 3.7% paraformaldehyde were imaged using a Nikon Eclipse Ti microscope and analysed using Fiji [27].

### 2.5 Microscopy

Bacterial samples were harvested by centrifugation, washed once with PBS and fixed in 4% paraformaldehyde. For phase-contrast microscopy, samples were mounted in 80% glycerol and imaged using a Nikon Ti Eclipse inverted microscope. Samples for transmission electron microscopy were fixed as above before additional fixation in 3% glutaraldehyde, 0.1 M cacodylate buffer. Fixed samples were then treated with 1% OsO_4_, dehydrated in ethanol and embedded in araldite resin. Embedded samples were sectioned at 85 nm on a Leica UC6 ultramicrotome, transferred onto coated copper grids, further stained with uranyl acetate and lead citrate and visualised using a FEI Tecnai BioTWIN TEM at 80 kV fitted with a Gatan MS600CW camera.

For fluorescence confocal microscopy, bacteria were grown in TY broth containing 500 nM HADA [28], labelled with 250 nM SNAP-Cell TMR-Star (New England Biolabs) and grown under anaerobic conditions for a further 60 min. Following labelling, cells were harvested at 8000×*g* for 2 min at 4 °C and washed twice in 1 ml ice cold PBS. Cells were resuspended in PBS and fixed in a 4% paraformaldehyde at room temperature for 30 min with agitation. Cells were washed three times in 1 ml ice cold PBS, immobilized by drying to a coverslip and mounted in SlowFade Diamond (Thermo Fisher Scientific). Images were captured using a Zeiss AiryScan confocal microscope.

## 3 Results

### 3.1 *C. difficile* produces a SpoVD homologue that is required for sporulation

The *C. difficile* R20291 genome encodes 10 putative penicillin-binding proteins (PBPs) (Table 2) and one predicted monofunctional transglycosylase (CDR20291_2283). In our previous transposon mutagenesis study only two of these, CDR20291_0712 and 0985, were identified as essential for growth *in vitro* [8]. However, five of the PBPs were required for formation of heat-resistant spores, including two with homology to the *B. subtilis* cortex specific PBP SpoVD, CDR20291_1067 and 2544. Of these only CDR20291_2544 has the C terminal PASTA domain that is characteristic of the *B. subtilis* sporulation-specific PBPs [18]. CDR20291_2544 (SpoVD_Cd_) shares 40.1% amino acid identity with *B. subtilis* SpoVD and has the same predicted overall organisation, with an N terminal predicted transmembrane helix, followed by a PBP dimerization domain (PF03717), a transpeptidase domain (PF00905) and the C terminal PASTA domain (PF03793). *spoVD* is located immediately downstream of CDR20291_2545 (Fig. 2A), encoding a protein with weak homology to *B. subtilis* FtsL (18.8% amino acid identity). Despite the weak similarity, the *C. difficile* and *B. subtilis* proteins are very similar in size (115 and 117 amino acids respectively), have a similar PI (9.57 and 9.63 respectively) and both have a high proportion of lysine residues (22.6% and 14.5% respectively). CDR20291_2545 and *spoVD*_*Cd*_ appear to be in an operon, with the promoter upstream of CDR20291_2545. In our earlier TraDIS screen, CDR20291_2545 was also found to be required for sporulation, although this may have been due to polar effects on *spoVD*_*Cd*_.

**Table 2:** Putative *C. difficile* PBPs.

*C. difficile* R20291 gene designation	Best *B. subtilis* strain 168 hit	Amino acid identity	Essential *in vitro*?
0712	PonA	27.3%	Yes
2544	SpoVD	40.1%	No but required for sporulation
1067	SpoVD	27.9% (PbpB 26.6%)	No but required for sporulation
1131	DacF	43.8%	No but required for sporulation
1318	PbpX	21.3% (PbpE 20.4%)	No
2048	DacF	31.5%	No but required for sporulation
0441	DacF	30.3%	No
0985	PbpA	21.2%	Yes
3056	PbpX	20.1%	No but required for sporulation
2390	DacB	27.5%	No

**Fig. 1:**
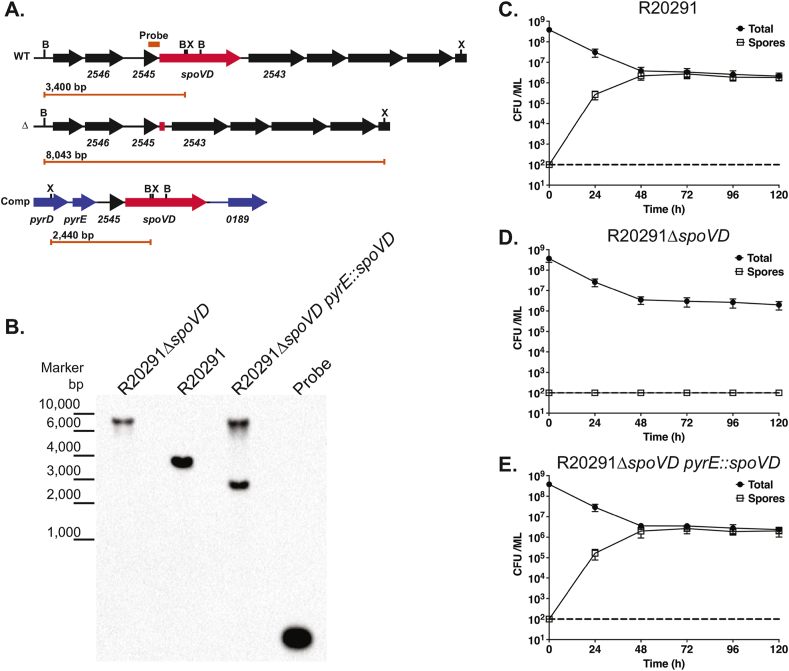
Sporulation requires SpoVD_Cd_. **A.** Genomic organisation of the native *spoVD*_*Cd*_ locus (WT), following deletion of the *spoVD*_*Cd*_ gene (Δ) and following complementation by insertion of R20291_2545 and *spoVD*_*Cd*_ between the *pyrE* and R20291_0189 genes (Comp). The locations of XmnI (X) and BsrGI (B) sites are indicated, as is the annealing site of the Southern blot probe. The length of the diagnostic restriction product containing the probe sequence is also shown below each locus diagram. **B.** Southern blot analysis of a *spoVD*_*Cd*_ mutant (R20291Δ*spoVD*), the wild type parental strain (R20291) and complemented strain (R20291Δ*spoVD pyrE*:*spoVD*). A DNA ladder is shown on the left hand side. The predicted fragment sizes and annealing site of the probe are shown in panel A. **C.-E.** Sporulation efficiencies of the wild type (**C.**), *spoVD*_*Cd*_ mutant (**D.**) and complemented strains (**E.**). Stationary phase cultures were incubated anaerobically for 5 days with samples taken daily to enumerate total colony forming units (CFUs) and spores, following heat treatment to kill vegetative cells. Experiments were performed in duplicate on biological triplicates with mean and standard deviation shown. The dotted horizontal line indicates the limit of detection of the experiment.

**Fig. 2:**
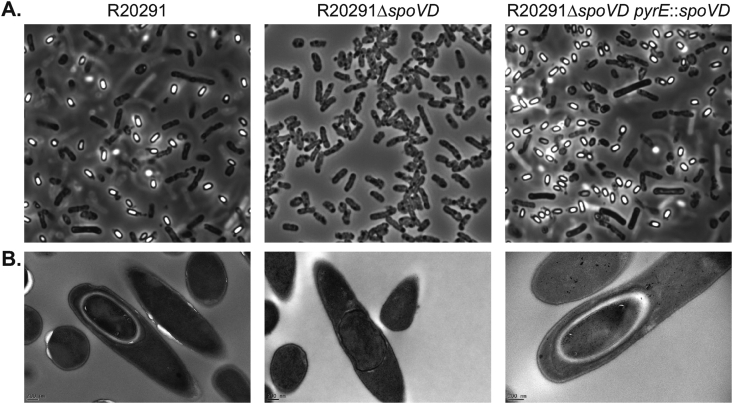
Microscopic analysis of sporulation. Phase-contrast light microscopy (**A.**) and negative stained TEM (**B.**) of the wild type parental strain (R20291), *spoVD*_*Cd*_ mutant (R20291Δ*spoVD*) and complemented strain (R20291Δ*spoVD pyrE*:*spoVD*). **A.** Cultures were imaged at day 5 of the sporulation assays shown in Fig. 1. Spores are visible as ovoid phase bright objects i, while vegetative cells are phase dark bacilli. **B.** TEM imaging of developing spores clearly shows normal spore development in R20291 and R20291Δ*spoVD pyrE*:*spoVD*; the densely stained core surrounded by a thick, largely unstained cortex layer. Cultures of R20291Δ*spoVD* contained no morphologically normal developing spores, although fully engulfed prespores without a cortex (example shown) were common.

**Fig. 3:**
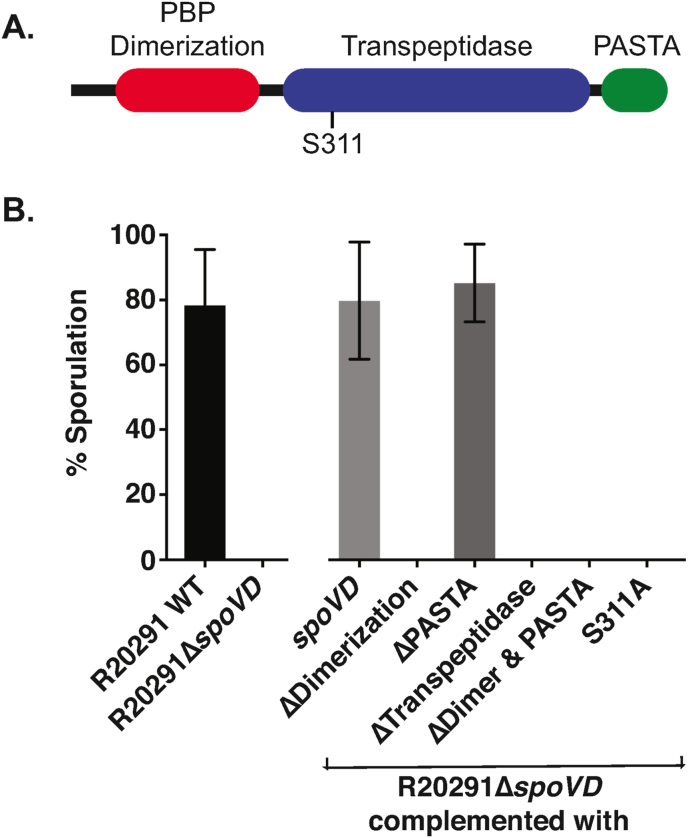
The contribution of SpoVD_Cd_ domains to sporulation. **A.** The domain organisation of SpoVD_Cd_ showing Pfam predictions [33]. **B.** Sporulation efficiency of R20291, R20291Δ*spoVD* and R20291Δ*spoVD* complemented *in trans* using plasmids expressing a series of mutant CLIP-SpoVDs under the control of a constitutive promoter: full-length CLIP-SpoVD_Cd_ (*spoVD*); CLIP-SpoVD_Cd_ lacking the PBP dimerization domain (ΔDimerization), PASTA domain (ΔPASTA), transpeptidase domain (ΔTranspeptidase) or both PBP dimerization and PASTA domains (ΔDimer & PASTA); CLIP-SpoVD_Cd_ lacking the active site nucleophile serine (S311A). Shown is the sporulation efficiency after 5 days in broth culture, expressed as number of spores as a percentage of total viable CFUs. Experiments were conducted in duplicate on biological triplicates and mean and standard deviations are shown.

To confirm a role in sporulation, we constructed a clean *spoVD*_*Cd*_ deletion by homologous recombination and then complemented this mutant by integrating the CDR20291_2545-*spoVD*_*Cd*_ cassette under the control of the native promoter into the chromosome between the *pyrE* and R20291_0189 genes (referred to here as R20291Δ*spoVD pyrE*:*spoVD*; Fig. 1A and B). We then analysed the ability of each strain to form heat-resistant spores. In our assay, a stationary phase culture of wild type R20291 gradually accumulated spores, accounting for 81% of the viable counts after 3 days (Fig. 1C). In the same assay R20291Δ*spoVD* formed no detectable spores, even after 5 days of incubation (Fig. 1D). Complementation completely restored sporulation to wild type levels (Fig. 1E). Examination by phase-contrast microscopy confirmed the presence of abundant mature phase bright spores in 5 day old cultures of wild type R20291 and the complemented strain R20291Δ*spoVD pyrE*:*spoVD* (Fig. 2A). In contrast no phase bright objects were observed in cultures of R20291Δ*spoVD*. When visualised at higher magnification using TEM of thin sections, no morphologically normal spores were observed in cultures of R20291Δ*spoVD* (Fig. 2B). Membrane-bound prespores were present, but these structures were irregular in shape and crucially lacked the cortex and protein coat layers seen in R20291 and the complemented strain developing spores. SpoVD_Cd_ is predicted to consist of 3 domains: a PBP dimerization domain, a transpeptidase domain and a PASTA domain (Fig. 3A). To identify which of these were required for viable spore formation, *CLIP-spoVD*_*Cd*_ was placed under the control of a constitutive promoter (P_*cwp2*_) in a *C. difficile* expression vector and a panel of mutants, lacking one or more of these domains, were constructed (Table 1). These plasmids were all transferred into R20291Δ*spoVD* and the ability of the expressed CLIP-SpoVD_Cd_ variant to restore sporulation was evaluated. Only proteins including both the dimerization and transpeptidase domains (SpoVD(ΔPASTA) or full-length SpoVD) restored normal sporulation (Fig. 3B), the PASTA domain was dispensable as observed previously in *B. subtilis* [18]. This observation was supported by TEM examination, with morphologically normal spores only observed when the full-length or SpoVD(ΔPASTA) proteins were expressed (not shown).

*B. subtilis* SpoVD, and the wider family of class B PBPs, share a conserved active site consisting of 3 non-contiguous motifs that are brought into close proximity in the folded enzyme, SxxK, SxN and KTG [29]. The first of these motifs contains the essential serine nucleophile. SpoVD_Cd_ has all three motifs, with Ser311 as the predicted nucleophile. SpoVD_Cd_ S311A supplied *in trans* was also incapable of complementing the sporulation defect observed in a *spoVD*_*Cd*_ deletion mutant (Fig. 3B), confirming a role for this residue in cortex synthesis.

### 3.2 Subcellular localisation of SpoVD_Cd_

**Fig. 4:**
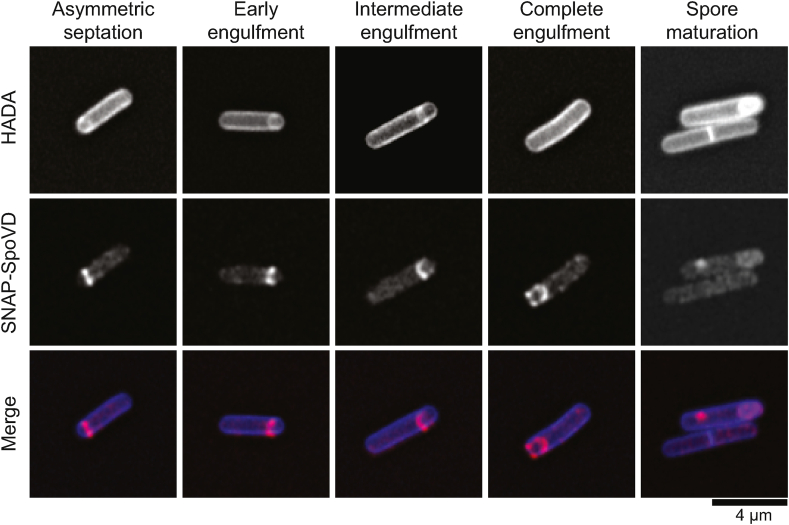
Subcellular localisation of SpoVD_Cd_. R20291 *SNAP-spoVD*_*Cd*_ was grown for 24 h in TY broth containing the fluorescent d-amino acid HADA (500 nM) to label *de novo* synthesised peptidoglycan. The bacteria were then further stained with SNAP-Cell TMR-Star (250 nM) to label SNAP-SpoVD_Cd_, fixed, mounted in SlowFade Diamond mountant and imaged using a Zeiss AiryScan confocal microscope. Shown are representative cells demonstrating the sequential stages of sporulation: asymmetric septum placement, early, intermediate and complete prespore engulfment respectively, and spore maturation.

To visualise the cellular localisation of SpoVD_Cd_, we fused the coding sequence for SNAP to the 5′ end of the *spoVD*_*Cd*_ gene and transferred this to the *C. difficile* genome in the native locus and under the control of the native promoter. SNAP was then labelled with the fluorescent reagent TMR-Start, while newly synthesised peptidoglycan was labelled with the fluorescent d-amino acid HADA [28]. Using Airyscan confocal microscopy we observed weak punctate fluorescence around the periphery of the cell, localizing to the asymmetric division septum once the cell had committed to sporulation (Fig. 4A). Fluorescence then tracked the asymmetric membrane through engulfment (Fig. 4B and C), eventually surrounding the prespore (Fig. 4D). Localisation of SNAP-SpoVD_Cd_ clearly preceded significant cortex synthesis as we visualised localisation around the spore without concomitant HADA accumulation (Fig. 4D). Following further spore maturation (Fig. 4E), accumulation of new HADA-labelled peptidoglycan co-localized with SNAP-SpoVD_Cd_.

## 4 Discussion

*C. difficile* is the most common cause of hospital acquired infection in the USA and Europe [30,31]. The formation of a robust spore form is crucial for transmission of infection between patients and for persistence and relapse following treatment [5]. However, despite their importance in *C. difficile* pathogenesis, we still know surprisingly little about the underlying molecular mechanisms of sporulation and germination, in part due to a lack of effective genetic tools until recently [32]. Much can be learned from the parallels with the well-studied but distantly related species *B. subtilis*, however there are significant differences in the sporulation pathways between the *Bacillales* and *Clostridiales* and even homologous proteins can play subtly different roles [[9], [10], [11]]. Here we have identified and characterised a *C. difficile* homologue of the *B. subtilis* spore cortex PBP SpoVD. We have confirmed that this protein is required for sporulation in *C. difficile*.

Bioinformatic analysis of the *C. difficile* genome identified 10 genes encoding proteins with significant homology to characterised *B. subtilis* PBPs. In a previous transposon mutagenesis screen we determined that only two of these are essential for growth *in vitro*, but five were required for formation of heat-resistant spores [8]. One of these (R20291_2544) encodes a protein sharing 40.1% amino acid identity with *B. subtilis* SpoVD. Despite this relatively weak homology, the two proteins share a similar overall domain organisation and are encoded in a similar genomic context. To determine if this protein played a role in *C. difficile* sporulation we constructed a clean deletion mutant that we found to be incapable of producing viable spores. Microscopic examination of this mutant allowed us to visualise fully engulfed prespores but these structures lacked any obvious cortex. This sporulation defect was fully complemented by integration of *spoVD*_*Cd*_ (and the upstream R20291_2545 and native promoter) in a distal chromosomal locus. These observations clearly demonstrated that SpoVD_Cd_ plays a crucial role in *C. difficile* sporulation and is required for the synthesis of cortex peptidoglycan. We then demonstrated that the sporulation defect in a *spoVD*_*Cd*_ mutant could be complemented by expression *in trans* of a mutant CLIP-SpoVD_Cd_ lacking the C terminal PASTA domain but that mutation of the PBP dimerization or transpeptidase domains resulted in a non-functional SpoVD_Cd_. This is in full agreement with previous *B. subtilis* studies that showed that the PASTA domain was dispensable for cortex synthesis [18]. By comparison with the *B. subtilis* sequence we were also able to putatively identify the active site nucleophile serine as S311 and confirmed this role by mutation to alanine, resulting in a non-functional SpoVD_Cd_.

It has been shown previously that *B subtilis* SpoVD localises to the asymmetric septum upon initiation of sporulation and ultimately to the developing spore following engulfment [17]. To visualise this process in *C. difficile* we generated a strain expressing SNAP-SpoVD_Cd_ under the control of the native promoter. Super-resolution fluorescence microscopy imaging of this strain showed clear localisation of SNAP-SpoVD_Cd_ to the asymmetric septum and to the developing spore. Intriguingly we also observed weak punctate fluorescence staining around the periphery of the mother cell. This could be indicative of mislocalisation as a result of the N terminal SNAP fusion or could suggest a broader role for SpoVD_Cd_ in vegetative cell peptidoglycan synthesis. To test this latter possibility, we examined the peptidoglycan composition of wild type and *spoVD*_*Cd*_ mutant cells but observed no obvious differences. However, given the enormous potential for redundancy with 10 encoded PBPs it is possible that small differences could be missed in this analysis.

Sporulation of *C. difficile* represents one of the most pressing clinical challenges in tackling recurrent disease in individual patients as well as preventing outbreaks in nosocomial settings. However, this cell differentiation pathway also represents a promising target for the development of *C. difficile*-specific therapeutics. Indeed it has been shown that inhibition of sporulation with the SpoVD-targeting cephamycin cefotetan prevents relapse in a mouse model of CDI [22]. In order to exploit this potential fully however, we must first develop a deeper understanding of both the complex regulatory processes that underpin sporulation as well as the function of the effector proteins that direct differentiation. Here we have identified and characterised a PBP that is absolutely required for production of viable spores and that we believe is a promising target for future therapeutics aimed at preventing recurrent disease and transmission.

## Funding

This work was supported by a PhD studentship from the Higher Committee for Education and Development in Iraq for Y.A.A. and by the 10.13039/501100000265Medical Research Council (P.O., grant number MR/N000900/1) and the 10.13039/100010269Wellcome Trust (J.A.K., grant number 204877/Z/16/Z). The funders had no role in study design, data collection and interpretation, or the decision to submit the work for publication.

## Author contributions

Y.A.A. and R.P.F. designed and coordinated the study. Y.A.A., P.O. and J.A.K. performed the experiments. R.P.F. wrote the paper with input from all co-authors.

## Declaration of competing interest

The authors declare that the research was conducted in the absence of any commercial or financial relationships that could be construed as a potential conflict of interest.

## References

[1] Lessa F.C., Mu Y., Bamberg W.M., Beldavs Z.G., Dumyati G.K., Dunn J.R. (2015). Burden of *Clostridium difficile* infection in the United States. N. Engl. J. Med..

[2] Smits W.K., Lyras D., Lacy D.B., Wilcox M.H., Kuijper E.J. (2016). *Clostridium difficile* infection. Nat Rev Dis Primers.

[3] Rupnik M., Wilcox M.H., Gerding D.N. (2009). *Clostridium difficile* infection: new developments in epidemiology and pathogenesis. Nat. Rev. Microbiol..

[4] He M., Miyajima F., Roberts P., Ellison L., Pickard D.J., Martin M.J. (2013). Emergence and global spread of epidemic healthcare-associated *Clostridium difficile*. Nat. Genet..

[5] Deakin L.J., Clare S., Fagan R.P., Dawson L.F., Pickard D.J., West M.R. (2012). The *Clostridium difficile spo0A* gene is a persistence and transmission factor. Infect. Immun..

[6] Dyer C., Hutt L.P., Burky R., Joshi L.T. (2019). Biocide resistance and transmission of *Clostridium difficile* spores spiked onto clinical surfaces from an American healthcare facility. Appl. Environ. Microbiol..

[7] Zhu D., Sorg J.A., Sun X. (2018). *Clostridioides difficile* biology: sporulation, germination, and corresponding therapies for *C. difficile* infection. Front Cell Infect Microbiol.

[8] Dembek M., Barquist L., Boinett C.J., Cain A.K., Mayho M., Lawley T.D. (2015). High-throughput analysis of gene essentiality and sporulation in *Clostridium difficile*. mBio.

[9] Fimlaid K.A., Bond J.P., Schutz K.C., Putnam E.E., Leung J.M., Lawley T.D. (2013). Global analysis of the sporulation pathway of *Clostridium difficile*. PLoS Genet..

[10] Paredes C.J., Alsaker K.V., Papoutsakis E.T. (2005). A comparative genomic view of clostridial sporulation and physiology. Nat. Rev. Microbiol..

[11] Underwood S., Guan S., Vijayasubhash V., Baines S.D., Graham L., Lewis R.J. (2009). Characterization of the sporulation initiation pathway of *Clostridium difficile* and its role in toxin production. J. Bacteriol..

[12] Paredes-Sabja D., Shen A., Sorg J.A. (2014). *Clostridium difficile* spore biology: sporulation, germination, and spore structural proteins. Trends Microbiol..

[13] Gilmore M.E., Bandyopadhyay D., Dean A.M., Linnstaedt S.D., Popham D.L. (2004). Production of muramic delta-lactam in *Bacillus subtilis* spore peptidoglycan. J. Bacteriol..

[14] Meador-Parton J., Popham D.L. (2000). Structural analysis of *Bacillus subtilis* spore peptidoglycan during sporulation. J. Bacteriol..

[15] Daniel R.A., Drake S., Buchanan C.E., Scholle R., Errington J. (1994). The *Bacillus subtilis spoVD* gene encodes a mother-cell-specific penicillin-binding protein required for spore morphogenesis. J. Mol. Biol..

[16] Fay A., Meyer P., Dworkin J. (2010). Interactions between late-acting proteins required for peptidoglycan synthesis during sporulation. J. Mol. Biol..

[17] Sidarta M., Li D., Hederstedt L., Bukowska-Faniband E. (2018). Forespore targeting of SpoVD in *Bacillus subtilis* is mediated by the N-terminal part of the protein. J. Bacteriol..

[18] Bukowska-Faniband E., Hederstedt L. (2015). The PASTA domain of penicillin-binding protein SpoVD is dispensable for endospore cortex peptidoglycan assembly in *Bacillus subtilis*. Microbiology.

[19] Peltier J., Courtin P., El Meouche I., Lemee L., Chapot-Chartier M.P., Pons J.L. (2011). *Clostridium difficile* has an original peptidoglycan structure with a high level of N-acetylglucosamine deacetylation and mainly 3-3 cross-links. J. Biol. Chem..

[20] Coullon H., Rifflet A., Wheeler R., Janoir C., Boneca I.G., Candela T. (2018). N-Deacetylases required for muramic-delta-lactam production are involved in *Clostridium difficile* sporulation, germination, and heat resistance. J. Biol. Chem..

[21] Diaz O.R., Sayer C.V., Popham D.L., Shen A. (2018). *Clostridium difficile* lipoprotein GerS Is required for cortex modification and thus spore germination. mSphere.

[22] Srikhanta Y.N., Hutton M.L., Awad M.M., Drinkwater N., Singleton J., Day S.L. (2019). Cephamycins inhibit pathogen sporulation and effectively treat recurrent *Clostridioides difficile* infection. Nat. Microbiol..

[23] Dupuy B., Sonenshein A.L. (1998). Regulated transcription of *Clostridium difficile* toxin genes. Mol. Microbiol..

[24] Kirk J.A., Fagan R.P. (2016). Heat shock increases conjugation efficiency in *Clostridium difficile*. Anaerobe.

[25] Cartman S.T., Kelly M.L., Heeg D., Heap J.T., Minton N.P. (2012). Precise manipulation of the *Clostridium difficile* chromosome reveals a lack of association between the tcdC genotype and toxin production. Appl. Environ. Microbiol..

[26] Ng Y.K., Ehsaan M., Philip S., Collery M.M., Janoir C., Collignon A. (2013). Expanding the repertoire of gene tools for precise manipulation of the *Clostridium difficile* genome: allelic exchange using *pyrE* alleles. PLoS One.

[27] Schindelin J., Arganda-Carreras I., Frise E., Kaynig V., Longair M., Pietzsch T. (2012). Fiji: an open-source platform for biological-image analysis. Nat. Methods.

[28] Kuru E., Tekkam S., Hall E., Brun Y.V., Van Nieuwenhze M.S. (2015). Synthesis of fluorescent D-amino acids and their use for probing peptidoglycan synthesis and bacterial growth in situ. Nat. Protoc..

[29] Sauvage E., Kerff F., Terrak M., Ayala J.A., Charlier P. (2008). The penicillin-binding proteins: structure and role in peptidoglycan biosynthesis. FEMS Microbiol. Rev..

[30] Aguado J.M., Anttila V.J., Galperine T., Goldenberg S.D., Gwynn S., Jenkins D. (2015). Highlighting clinical needs in *Clostridium difficile* infection: the views of European healthcare professionals at the front line. J. Hosp. Infect..

[31] Magill S.S., Edwards J.R., Bamberg W., Beldavs Z.G., Dumyati G., Kainer M.A. (2014). Multistate point-prevalence survey of health care-associated infections. N. Engl. J. Med..

[32] Shen A. (2019). Expanding the *Clostridioides difficile* genetics toolbox. J. Bacteriol..

[33] El-Gebali S., Mistry J., Bateman A., Eddy S.R., Luciani A., Potter S.C. (2019). The Pfam protein families database in 2019. Nucleic Acids Res..

[34] Stabler R.A., He M., Dawson L., Martin M., Valiente E., Corton C. (2009). Comparative genome and phenotypic analysis of *Clostridium difficile* 027 strains provides insight into the evolution of a hypervirulent bacterium. Genome Biol..

[35] Purdy D., O'Keeffe T.A., Elmore M., Herbert M., McLeod A., Bokori-Brown M. (2002). Conjugative transfer of clostridial shuttle vectors from *Escherichia coli* to *Clostridium difficile* through circumvention of the restriction barrier. Mol. Microbiol..

[36] Fagan R.P., Fairweather N.F. (2011). *Clostridium difficile* has two parallel and essential Sec secretion systems. J. Biol. Chem..

[37] Pereira F.C., Saujet L., Tome A.R., Serrano M., Monot M., Couture-Tosi E. (2013). The spore differentiation pathway in the enteric pathogen *Clostridium difficile*. PLoS Genet..

